# A major yellow rust resistance QTL on chromosome 6A shows increased frequency in recent Norwegian spring wheat cultivars and breeding lines

**DOI:** 10.1007/s00122-023-04397-9

**Published:** 2023-07-01

**Authors:** Min Lin, Jon Arne Dieseth, Muath Alsheikh, Ennian Yang, Josef Holzapfel, Friederike Schürmann, Laura Morales, Sebastian Michel, Hermann Buerstmayr, Sridhar Bhavani, Morten Lillemo

**Affiliations:** 1 Present Address: Department of Plant Sciences, Norwegian University of Life Sciences, Post Box 5003, 1432 Ås, Norway; 2 Graminor AS, Hommelstadvegen 60, 2322 Ridabu, Norway; 3 Crop Research Institute, Sichuan Academy of Agricultural Sciences, Chengdu, 610066 Sichuan China; 4 Secobra Saatzucht GmbH, Lagesche Str. 250, 32657 Lemgo, Germany; 5 Institute of Biotechnology in Plant Production, University of Natural Resources and Life Sciences Vienna, 3430 Tulln, Austria; 6 International Maize and Wheat Improvement Center (CIMMYT), 56237 El Batan, Texcoco, Estado de Mexico Mexico

## Abstract

**Key message:**

A major yellow rust resistance QTL, *QYr.nmbu.6A*, contributed consistent adult plant resistance in field trials across Europe, China, Kenya and Mexico.

**Abstract:**

*Puccinia striiformis* f. sp. *tritici*, causing wheat yellow rust (YR), is one of the most devastating biotrophic pathogens affecting global wheat yields. Owing to the recent epidemic of the *PstS10* race group in Europe, yellow rust has become a reoccurring disease in Norway since 2014. As all stage resistances (ASR) (or seedling resistances) are usually easily overcome by pathogen evolution, deployment of durable adult plant resistance (APR) is crucial for yellow rust resistance breeding. In this study, we assessed a Nordic spring wheat association mapping panel (*n* = 301) for yellow rust field resistance in seventeen field trials from 2015 to 2021, including nine locations in six countries across four different continents. Nine consistent QTL were identified across continents by genome-wide association studies (GWAS). One robust QTL on the long arm of chromosome 6A, *QYr.nmbu.6A,* was consistently detected in nine out of the seventeen trials. Haplotype analysis of *QYr.nmbu.6A* confirmed significant QTL effects in all tested environments and the effect was also validated using an independent panel of new Norwegian breeding lines. Increased frequency of the resistant haplotype was found in new varieties and breeding lines in comparison to older varieties and landraces, implying that the resistance might have been selected for due to the recent changes in the yellow rust pathogen population in Europe.

**Supplementary Information:**

The online version contains supplementary material available at 10.1007/s00122-023-04397-9.

## Introduction

Yellow rust (YR) (syn. stripe rust), caused by *Puccinia striiformis* f. sp. *tritici* (*Pst*), is one of the most devastating biotrophic diseases on wheat (*Triticum aestivum* L.), and can cause up to 80% regional yield losses under weather conditions beneficial for epidemics (Wellings 2011). Chemical application remains an efficient method for controlling yellow rust damages when susceptible varieties are grown, but the cost of chemical products and the risk of causing fungicide resistant races, as well as the negative influence on environment, are still of concerns.

Deployment of resistant wheat cultivars serves as a both economically and environmentally friendly control method. Generally, YR resistance genes can be classified into two major classes: (1) “all stage resistance” (ASR), also known as seedling resistance, usually counters one or only few races; and (2) adult plant resistance (APR) usually shows effect at the adult plant stage and combats more races in contrast with ASR genes (Chen et al. 2014; Bouvet et al. 2022a). As a typical biotrophic pathogen, *Pst* interacts with wheat ASR genes following the gene-for-gene model (Flor 1956), whereby the resistance is achieved by recognition of the avirulence gene (*Avr*) of the pathogen by the corresponding resistance gene in the host. As such mechanism requires the match of both host resistance (ASR) gene, and pathogens` avirulence gene (*Avr*), the effects are usually qualitative and easy to overcome by the rapid evolution of highly dynamic pathogen populations (McDonald and Linde 2002). In contrast to the ASR genes which are only effective in the short term, APR genes have shown better durability. Despite significant progress made in identifying and characterizing APR genes in plants, the understanding of their underlying mechanisms remains limited compared to that of the seedling resistance genes (ASR).

Thanks to the research efforts spanning over decades, more than 300 wheat YR resistance related quantitative trait loci (QTL) have been mapped, and around 80 of them have been designated as *Yr* genes, of which the majority were ASRs (Jamil et al. 2020; McIntosh et al. 2020). However, very few have been characterized at the DNA sequence level (McIntosh et al. 2020). To date, among the plant disease resistance genes being cloned, most genes encode nucleotide-binding leucine-rich repeat (NLR) domains (Wulff and Krattinger 2022). As reviewed lately by Bouvet et al. (2022a), 90% of the cloned rust resistance genes harbor NLR domains and most of them are ASR genes. For example, *Yr7* (Marchal et al. 2018), *Yr10* (Liu et al. 2014), *Yr15* (Klymiuk et al. 2018), *YrAS2388R* (Zhang et al. 2019), *YrU1* (Wang et al. 2020) and *Yr27* (Athiyannan et al. 2022) are YR ASR genes being cloned so far (Hafeez et al. 2021). Of these, *Yr15* is the only exception by encoding tandem kinase-pseudokinases (TKPs) and not containing any NLR domain (Klymiuk et al. 2018). Surprisingly, different from the other NLR coding genes, *Yr15* also exhibits broad spectrum resistance against a worldwide collection of isolates (Klymiuk et al. 2018). Additionally, a recent study also showed that *Yr15*/*YrG303*/*YrH52* were encoded by a single locus wheat tandem kinase 1 (*Wtk1*) (Klymiuk et al. 2020).

However, the mechanisms of the APR genes seem more obscure, as only a small number of APR genes have been cloned, and each of them encodes different proteins involved in different biochemical pathways (Bouvet et al. 2022a). Krattinger et al. (2009) characterized the wheat rust APR gene *Yr18*/*Lr34/Pm38,* which encodes a putative ATP-binding cassette (ABC) transporter and confers broad spectrum resistance not only against yellow rust, but also two other biotrophic pathogens causing wheat leaf rust and powdery mildew (Krattinger et al. 2009). Another example, *Yr36* encodes a wheat kinase START 1 (WKS1) (Fu et al. 2009), which contributes to plant cell death but the response takes a few days longer than normal hypersensitive reaction (HR) (Gou et al. 2015). As a result, it limits pathogen growth and confers a broad-spectrum resistance against various *Pst* races (Fu et al. 2009). Moore et al. (2015) also cloned an APR gene *Yr46*/*Lr67*/*Pm46*/*Sr55*, which confers pleiotropic effect against all three rust diseases and powdery mildew. Nevertheless, different from the other known pleiotropic gene *Yr18*, *Yr46* encodes a hexose transporter, which reduces pathogen growth by alterations in glucose uptake (Moore et al. 2015).

To achieve durable resistance, breeders have to either pyramid multiple ASR genes or/and exploit APR genes in the breeding materials, since single ASR genes can easily be overcome in a few years after the cultivar release, due to virulence changes in the pathogen populations. As an example, the new yellow rust race group *PstS10* quickly replaced the existing European YR population since its first arrival in 2011 (Hovmøller et al. 2016). As a consequence, the most widely grown spring wheat varieties in Norway were rendered susceptible (Abrahamsen et al., 2017). It is crucial to look for durable YR resistance resources and improve the resistance in the local wheat germplasm. In the current study, we assessed the YR resistance of a Nordic spring wheat association mapping panel under field conditions. Seventeen trials were conducted at nine locations across six countries (Norway, Austria, Germany, China, Mexico, and Kenya), covering four different continents where the dominant races are likely to be different (Ali et al. 2017). The main objective of this study was to identify adult plant YR resistance QTL which could potentially be durable and effective across a wide range of environments. Subsequently, to validate the detected QTL in independent germplasm and provide KASP markers to breeders, which can be utilized in marker-assisted selection for improving YR resistance in breeding programs.

## Materials and methods

### Plant material and genotyping

The NMBU spring wheat panel (MASBASIS) used in the present study was previously described by Lin et al. (2022). Briefly, this association panel contains 301 spring wheat lines which covers the most currently and historically important wheat cultivars and breeding lines in the Nordic region. In addition to that, a few international lines from CIMMYT and China were also included in the panel. The division of the population structure followed the origin of the lines, with one subpopulation mainly including lines originating from the Nordic region while the other subpopulation contained other international lines. The panel was genotyped by the TraitGenetics 25 K wheat SNP chip, which comprised a total of 22,205 markers, including 14,455 markers from the 90 K Infinium array, 2949 markers from the 35 K Wheat Breeders array, 5536 new markers from the 135 K Axiom array, and 265 gene- and trait-specific markers obtained from the literature. As described by Lin et al. (2022), some KASP and SSR markers were also included in the genotype file. Markers with allele frequency less than 5% were filtered out resulting in 18,578 markers for the genetic analyses.

In addition, a panel of around 300 spring wheat breeding lines from Graminor was also tested in four environments in Norway from 2018 to 2019, one environment in 2021 at Tulln, Austria and one environment in 2021 at Lemgo, Germany. The validation panel was genotyped by the same TraitGenetics 25 K SNP chip and was used for validating the haplotype effect of the *QYr.nmbu.6A* QTL.

## Field trials

In total, seventeen field trials were conducted in six countries at nine locations for the NMBU spring wheat panel (three locations in Norway, one in Germany, one in Austria, one in Kenya, one in Mexico and two locations in China). The panel was evaluated for adult plant resistance to yellow rust in hillplot trials together with the resistant check `Saar` and susceptible checks ′MS273-150′, ′Avocet YrA′ and ′GN12737`, at the Vollebekk field station in Ås, Norway for five years from 2015 to 2019, referred to hereafter as Vb2015, Vb2016, Vb2017, Vb2018 and Vb2019 experiments, respectively. In addition, YR phenotyping was also conducted in 2016 in Staur, Norway (St2016) and 2019 in Holmestrand, Norway (Hs2019). Alpha lattice designs with two replicates were utilized for conducting the hillplot trials. The susceptible check ′Avocet YrA` was planted as spreader rows at the edges of the field trial, followed by 3 rows of the moderately resistant cultivar ′Bastian′ to prevent border effects. Field trials in Norway were naturally infected by YR, except in 2018 at Vollebekk where heavily infected seedlings by race “Warrior –” were planted in empty spaces of the spreader rows to inoculate the spreader. When needed, mist irrigation was applied in some of the trials conducted at Vollebekk in order to create good conditions for infection. Visual scorings of the disease severity (%) were undertaken when the susceptible check ′Avocet YrA′ reached 70–100%.

A trial of the NMBU spring wheat panel was conducted in 2020 at Feldkirchen (FK2020) in the south of Germany, while the validation panel was tested in 2021 at Lemgo (Le2021) in western Germany. Non-replicated trials were surrounded with highly YR-susceptible wheat genotypes as spreaders and inoculated with a mixture of the current aggressive and widespread YR isolates obtained from Dr. Kerstin Flath, Julius Kühn-Institute in Germany, the federal official institution for plant protection.

Trials in Austria were conducted at Tulln, in 2020 (Tu2020) for the NMBU spring wheat panel and in 2021 (Tu2021) for the validation panel. Randomized complete block design with two replicates were utilized for the field trials. YR susceptible cultivar ′Remus′ was used as spreader row inoculated with YR isolates mixture collected from the previous season.

Trials in China were conducted at the Xindu and Pixian (Sichuan Province) research stations for three years from 2019 to 2021 (XD2019, PX2019, XD2020, PX2020, XD2021, and PX2021) growing seasons. Field trials were conducted in randomized complete blocks with two replications. About 30 seeds were sown in rows of 1.0 m long and 0.25 m apart. ′SY95-71′ was planted every 20 rows as susceptible checks and surrounding the nursery for increasing stripe rust pressure. The surrounding spreaders were artificially inoculated with a mixture of currently prevalent *Pst* races in China, including CYR32, CYR33, CYR34, and G22-14.

A trial with the NMBU spring wheat panel was conducted at CIMMYT in Toluca, Mexico in 2021 with augmented design, where ′Apav#1′ and ′PBW343′ were used as susceptible checks and replicated 25 times. The field trial was artificially infected by predominant Mexican *P*st race having avirulence/virulence: *Yr1*, *4*, *5a*, *10*, *15*, *24*, *26*, *5b*, *Poll*/*Yr2*, *3*, *6*, *7*, *8*, *9*, *17*, *27*, *31*, *A* (Randhawa et al. 2018). During the trial, a variety of susceptible genotypes were used in the infector rows to test for resistance to YR. For the spread of YR, a mixture of ′PBW343′, ′Morocco′, ′Murga′, ′NANA′ lines from ′Avocet′/′Atilla′ crosses, ′Yr24′/′Avocet′ and ′Yr26′/′Avocet′ were used. The spreader rows were inoculated with around 4–5 weeks post germination, and the process was repeated three times to ensure good infection of spreader rows.

In 2021, field trial evaluation was conducted at the stem rust phenotyping platform at KALRO (Kenya Agricultural Livestock Research Organization), Njoro, Kenya with partially replicated design. 20 lines were replicated two times and three checks (′CACUKE′, ′PBW343′ and ′KINGBIRD′) were replicated 10 times. The yellow rust trial in Kenya was naturally infected predominant races present at KALRO, Njoro belonged to race *PstS2* and *PstS11* (Hovmøller et al. 2022) based on GRRC race analysis. However, presence of other races in minor frequencies cannot be ruled out.

## Statistical analysis

For each trial, corrections of the random block effects and calculations of the mean YR score (Best Linear Unbiased Estimators, BLUEs) of each line were done using Proc MIXED in SAS v.9.4 (SAS Institute Inc.). As the YR scores were not normally distributed (Fig. S1), data transformations were optimized by using the function transformTukey implemented in the R package ′ rcompanion′ (Mangiafico, 2021), where

$${\text{YR transformed}} = {\text{YR }}^{ \wedge } 0.3$$

The ′ rcorr′ function, implemented in the R package Hmisc (Harrell 2019), was used for calculating the pair-wise Pearson correlation coefficients of the original YR score (%). “mmer” function implemented in R package “sommer” (Covarrubias-Pazaran 2016) was used to calculate variance components, where genotype and environment were set as random effect. Broad sense heritability was calculated separately for all trials, trials within Europe and trials within China with formula ${h}^{2}={\sigma }_{g}^{2}/({\sigma }_{g}^{2}+{\sigma }_{E}^{2}/t+{\sigma }_{e}^{2}/t)$, where ${\sigma }_{g}^{2}$ is the genetic variance, ${\sigma }_{E }^{2}$ is the environment variance, ${\sigma }_{e}^{2}$ is the error variance, t is the number of trials.

## Association and bioinformatic analysis

Calculation of the pairwise Linkage disequilibrium (LD) for markers on each chromosome was achieved by using the full-matrix option in TASSEL (Bradbury et al. 2007). Association analyses were conducted by using the “FarmCPU” model implemented by the R package GAPIT3 (Liu et al. 2016; Wang and Zhang 2021). Both the original and the transformed YR scores were used as phenotypic inputs for the GWAS analysis. Markers were considered as significant when associated markers met the threshold of FDR adjusted * P* value < 0.05. As described by Lin et al., (2022), two databases https://triticeaetoolbox.org and http://www.cerealsdb.uk.net were used to obtain marker sequences, and physical map positions of markers were obtained by the wheat reference genome IWGSC RefSeq v1.0 (International Wheat Genome Sequencing et al. 2018) database https://urgi.versailles.inra.fr/blast/?dbgroup=wheat_iwgsc_refseq_v1_chromosomes&program=blastn. Significant markers within 15 Mbp region were considered belonging to the same QTL as long as significant markers were located in the same LD block (*R*^2^ > 0.5, *D*′ > 0.8). Quantile–Quantile (QQ) plots of the p-values using both original YR scores and transformed YR scores were inspected and compared. Sequence comparisons with the wheat pan genome project (Walkowiak et al. 2020) were achieved by database https://galaxy-web.ipk-gatersleben.de/. The Confidence gene annotations (HighConfidenceGenesv1.1), and their corresponding functional annotations obtained from https://urgi.versailles.inra.fr/jbrowseiwgsc/gmod_jbrowse, were used for identification of candidate genes located within the interval of ± 1 Mbp of the most significant marker.

## Haplotype analysis

Among all consistent QTL detected in this study, *QYr.nmbu.6A* was found to be most significant and consistent, and was therefore selected for further haplotype analysis. We observed slight variation in the physical interval of *QYr.nmbu.6A* QTL that shifted from 598 to 610 Mbp to 610–612 Mbp when the transformed disease scores were used as phenotypic input instead of the original disease scores, however, marker *GENE-4021_496* was significantly detected no matter which input data was used. Therefore, the three most significant markers *AX-158600183*, *GENE-4021_496* and *AX-95182345* spanning the whole QTL interval of 598–612 Mbp were selected to construct the haplotypes. Pairwise comparison of YR scores (%) between haplotypes in each environment were achieved by using the Wilcoxon test by the function “stat_compare_means” in R package “ggpubr” (Kassambara, 2020).

## Development of KASP markers for *QYr.nmbu.6A* and tracing back the origin of the resistant source

To trace back the origin of the *QYr.nmbu.6A* resistant source, a collection of 114 wheat cultivars were sourced for KASP genotyping, which mainly consisted of old Nordic spring wheat cultivars and landraces in the pedigree of lines carrying the resistant haplotype in our GWAS panel. All three significant markers used for *QYr.nmbu.6A* haplotype analysis and markers with high LD of those three markers were selected for KASP genotyping (Table S1). The primers and sequence polymorphisms of KASP markers were obtained from the database https://www.cerealsdb.uk.net/cerealgenomics/CerealsDB/axiom_download.php (Table S1). Pedigree information of the cultivars and breeding lines were obtained from the wheat pedigree database (http://wheatpedigree.net/), the Nordic Baltic Genebanks Information System (GeNBIS) (https://www.nordic-baltic-genebanks.org/gringlobal/) and GRIN-Global (https://npgsweb.ars-grin.gov/gringlobal/search).

## Stacking resistant alleles

Markers from the nine common QTL which were significantly identified across continents were selected for stacking resistant alleles (Table S2). Resistant alleles were defined by comparing the mean of disease severities of trials in Europe (Fig. S2) and mean of disease severities of trials in China (Fig. S3) for each allele through the Wilcoxon test. As no significant difference was found between alleles of marker *AX-109326625* for both means of trials in Europe and China, this marker was excluded from the allele stacking analysis.

## Results

### Phenotypic variations

**Fig. 1:**
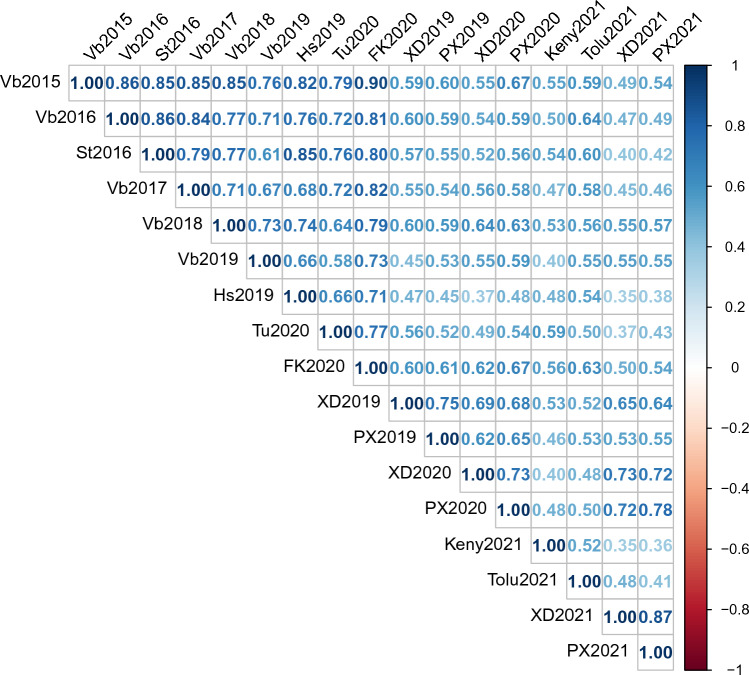
Pearson correlation coefficients for yellow rust severity (%) from all 17 field trials of the NMBU spring wheat panel, Vb: Vollebekk, Norway; St: Staur, Norway; Hs: Holmenstrand, Norway; FK: Feldkirchen, Germany; PX: Pixian, China; XD: Xindu, China; Tu: Tulln, Austria, Keny: Kenya, Tolu: Toluca, Mexico

Variations in yellow rust resistance were observed in all tested seventeen environments (Fig. S1). However, Fig. S1 also showed that the phenotypic distributions were right skewed. In general, trials in Europe showed lower disease pressure compared to trials in China, Mexico and Kenya. Especially for trials in Staur 2016, Vollebekk 2016, Vollebekk 2017, Holmestrand 2019 and Tulln 2020, where over 85% of the lines had disease severities less than 5%. Data transformation could improve the normality of the YR scores, however, YR scores of the most resistant lines which showed complete resistance (0% YR scores) remained unchanged (Fig. S4). According to Fig. 1, the YR scores were highly correlated among all tested environments regardless of the testing location, country or continent (*r* > 0.35, *p* < 0.00001). The correlation coefficients of YR scores between trials within Europe (Norway, Germany and Austria) varied from 0.58 to 0.90, while between trials within China varied from 0.53 to 0.87 (Fig. 1). However, relatively lower correlation coefficients were observed between trials from different continents (Fig. 1), for example between Asia and Europe (0.35–0.67), between North America and Europe (0.50–0.64), between Africa and Europe (0.40–0.59), between Asia and North America (0.41–0.53), and between Asia and Africa (0.35–0.53). The overall broad sense heritability of YR scores was 0.88, heritability within trials in Europe was 0.90, and heritability within trials in China was 0.90.

## Association analysis

As shown by the QQ plots, the significance of the markers from the analysis using the original YR scores as inputs was higher in 10 out of the 17 tested environments (Fig. S5). In addition, the original YR scores fitted the model almost as good as the transformed YR scores for the remaining environments (Fig. S5). Therefore, GWAS results of this study were based on the original YR scores as inputs.

In total, 91 marker trait associations (MTAs) were detected by GWAS analysis in this study (Table S3) and 19 consistent QTL were captured in at least two different environments, on chromosomes 1B, 2A, 2B, 2D, 3A, 3B, 3D, 4A, 4D, 5A, 5B, 6A, 6B, 7A and 7B (Table 1, Fig. S6). Among which, we identified nine QTL across trials from at least two continents. The most significant and consistent common QTL across continents was *QYr.nmbu.6A,* which was located at 598–612 Mbp and being significantly detected by six trials in Norway, one trial in Austria and two trials in China. The -log10(p) value of *QYr.nmbu.6A* ranged from 6.82 to 37.64 and was found to account for up to 90.4% of the phenotypic variation. In addition, *QYr.nmbu.1B,* on chromosome 1B, was detected by trials in China and Germany, of which both significant markers *AX-158521173* and *Tdurum_contig65853_242* were mapped to the similar physical map position at 629 Mbp, and phenotypic variation explained varied from 1.4 to 5.8%. *QYr.nmbu.2A.2* was located at 717–729 Mbp on chromosome 2A, which was identified by trials in Kenya and China in 2021. Another common QTL across Kenya and China was *QYr.nmbu.2D*, which was likely caused by the photoperiod response gene *Ppd-D1*, as the significant marker of this QTL was the KASP marker *PpdDD001* (Rasheed et al. 2016). *QYr.nmbu.4A* was a QTL common between China and Europe, which was identified by one trial in China and one trial in Germany. Both significant markers of this QTL were located at 631 Mbp on chromosome 4A with − log10(p) value ranging from 5.01 to 5.62. The significant marker *AX-94680941* of QTL *QYr.nmbu.4D*, located at 439 Mbp on chromosome 4D, was consistently detected by one trial in China and one trial in Germany. Another common QTL between China and Europe was the QTL *QYr.nmbu.5A.2,* located at 503–508 Mbp on chromosome 5A (-log10(*p*): 4.52–8.38)*,* which was identified by one trial in Norway and two trials in China, explaining phenotypic variation up to 3.3%. Two common QTL on chromosome 5B were also identified as significant across China and Europe. The first one was *QYr.nmbu.5B.1*, which was located at 515–518 Mbp, while the second QTL *QYr.nmbu.5B.2* was located at 546 Mbp, the phenotypic variation explained by the QTL ranged from 1.0 to 8.3% and 5.5 to 7.4%, respectively.

**Table 1:** Consistent yellow rust QTL identified in the NMBU spring wheat panel tested in seventeen trials, at nine locations from 2015 to 2021. Vb: Vollebekk, Norway; St: Staur, Norway; Hs: Holmestrand, Norway; FK: Feldkirchen, Germany; XD: Xindu, China; PX: Pixian, China, Tu: Tulln, Austria, Keny: Kenya, Tolu: Toluca, Mexico. Physical positions were based on blastn result against reference genome IWGSC RefSeq v1.0 (International Wheat Genome Sequencing et al. 2018)

QTL	Chromosome	Trials	Total number of MTAs	Flanking markers	Position (Mbp)	− log10 (p)	Reference
*QYr.nmbu.1B*	1B	PX 2020, FK 2020, XD2021	2	AX-158521173 and Tdurum_contig65853_242	629	4.85–8.37	Tehseen et al. (2020)
*QYr.nmbu.2A.1*	2A	Vb 2016, Vb 2017 FK 2020	3	AX-94639471 and RAC875_c4015_2175	52–58	4.60–5.32	This study
*QYr.nmbu.2A.2*	2A	Keny2021, XD2021	2	wsnp_Ex_c16627_25162391 and AX-94482613	717–729	5.42–6.04	Tehseen et al. (2020); Yuan et al. (2020)
*QYr.nmbu.2B.1*	2B	XD2021, PX2021	2	AX-94833805 and BS00067907_51	711–722	5.94–7.12	Wu et al. (2020a)
*QYr.nmbu.2D*	2D	XD 2019, XD 2020, Keny 2021	1	PpdDD001	34	4.67–5.70	This study
*QYr.nmbu.3A.1*	3A	PX 2020,PX2021	1	AX-158577637 and AX-158577605	7	5.07–6.88	Yao et al. (2020); Rosewarne et al. (2013); Long et al. (2019); Bouvet et al. (2022b)
*QYr.nmbu.3A.2*	3A	Vb 2015, Vb 2017, Hs 2019, FK 2020	3	AX-94458766 and wsnp_JD_c5699_6859527	479–484	5.58–10.37	Bokore et al. (2021)
*QYr.nmbu.3B*	3B	St 2016, Vb 2017, Vb 2018, Hs 2019, Tu 2020	3	AX-94390743 and AX-158579096	818–819	5.00–17.51	Wu et al. (2020a)
*QYr.nmbu.3D*	3D	Hs 2019, St 2016	1	AX-94580748	610	7.18–19.49	This study
*QYr.nmbu.4A*	4A	St2016, XD2021	2	Kukri_c33315_159 and Kukri_rep_c109463_264	631	5.01–5.62	This study
*QYr.nmbu.4D*	4D	PX 2019, FK 2020	1	AX-94680941	439	6.14–6.86	This study
*QYr.nmbu.5A.1*	5A	Vb 2016, Vb 2018	1	Tdurum_contig59338_1902	489	5.92–8.51	This study
*QYr.nmbu.5A.2*	5A	St 2016, PX 2020, XD 2020	3	AX-109326625 and TA004548-0753	503–508	4.52–8.38	This study
*QYr.nmbu.5B.1*	5B	Vb 2017, Vb 2018, XD 2019, FK 2020, PX 2020	2	AX-108953209 and wsnp_Ku_c14202_22436656	515–518	5.05–8.85	Tehseen et al. (2020) Wu et al. (2020b)
*QYr.nmbu.5B.2*	5B	Vb 2019, XD 2020	1	Fcp001	546	10.52–10.93	This study
*QYr.nmbu.6A*	6A	Vb 2015, Vb 2016, Vb 2017, Vb 2019, St 2016, Hs 2019, PX 2019, XD 2019, Tu 2020	4	AX-158600183 and AX-95182345	598–612	6.82–37.64	Bouvet et al. (2022b); Shahinnia et al. (2022); Kale et al. (2022); Beukert et al. (2020)
*QYr.nmbu.6B*	6B	St 2016, Vb 2018, Hs 2019	7	BS00022823_51 and Tdurum_contig43872_549	94–113	5.12–9.83	Yr78 (Dong et al. 2017)
*QYr.nmbu.7A*	7A	Vb 2016, Vb 2019, FK 2020	2	Kukri_c18148_913 and RFL_Contig2814_604	724–731	4.57–6.42	Tehseen et al. (2020)
*QYr.nmbu.7B*	7B	Hs 2019, Tu 2020	1	Kukri_c14766_484	62	5.55–7.70	Yao et al. (2020)

Moreover, eight QTL were specific to environments in Europe. *QYr.nmbu.2A.1* was identified on the short arm of chromosome 2A (52–58 Mbp), which was significantly detected in two trials in Norway and one trial in Germany (Table 1). *QYr.nmbu.3A.2,* located at 479–484 Mbp on chromosome 3A, was identified as significant by three trials in Norway, in three different years, and the trial in Germany. Additionally, *QYr.nmbu.3B* was significantly detected by trials in Norway in three years and one trial in Austria. This QTL located at 818–819 Mbp on the physical map of chromosome 3B, but explained only 0.05–0.58% of the phenotypic variation. The marker *AX-94580748*, located at 610 Mbp on chromosome 3D, was consistently detected as significant in both trial St2016 and Hs2019 in Norway, with − log10(p) values varying from 7.18 to 19.49 and up to 1.9% explained the phenotypic variation. Another single marker *Tdurum_contig59338_1902*, located at 489 Mbp on chromosome 5A, was also significantly detected in two trials in Norway in both 2016 and 2018, which acted as a major QTL explaining phenotypic variation up to 31.9%. *QYr.nmbu.6B,* which was located at 94–113 Mbp on chromosome 6B, was identified as significant QTL in all three different locations in Norway but in different years. *QYr.nmbu.7A* was significantly detected by both years 2016 and 2019 at Vollebekk, Norway, and also the trial in Feldkirchen, Germany in 2020. The last Europe specific QTL, *QYr.nmbu.7B,* was located at 62 Mbp on chromosome 7B based on the consistent detection of marker *Kukri_c14766_484* in one trial in Norway and one trial in Austria. Two consistent QTL were detected private to trials in China, which were *QYr.nmbu.2B.1* and *QYr.nmbu.3A.1* (Table 1)*. QYr.nmbu.2B.1* (711–722 Mbp) was detected from both locations in China but only in 2021, while *QYr.nmbu.3A.1* (7 Mbp) was identified by the trials in Pixian 2020 and 2021.

## Haplotype analysis

**Fig. 2:**
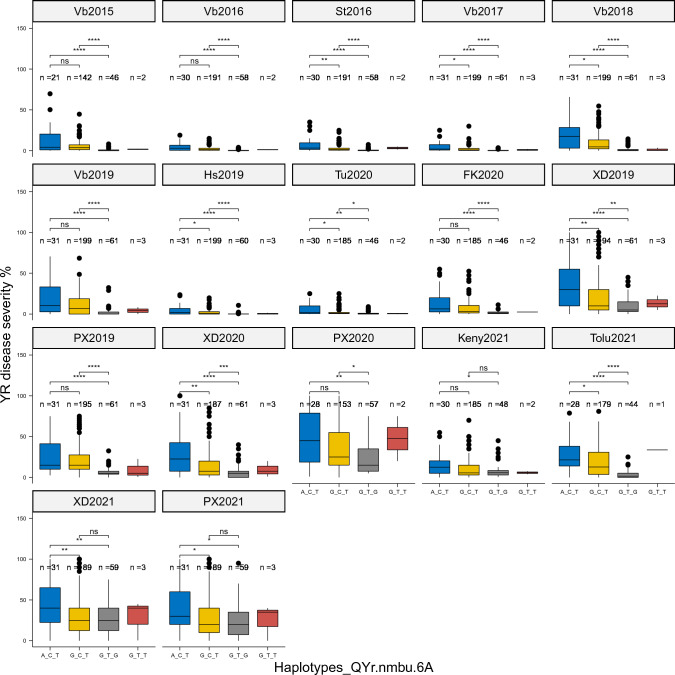
Haplotype analysis of QTL *QYr.nmbu.6A* on the NMBU spring wheat panel of trials from seventeen environments. Vb: Vollebekk, Norway; St: Staur, Norway; Hs: Holmestrand, Norway; FK: Feldkirchen, Germany; Tu: Tulln, Austria; XD: Xindu, China; PX: Pixian, China; Keny: Kenya; Tolu: Toluca, Mexico; Differences in yellow rust (YR) severity (%) between haplotypes were determined by the Wilcoxon test. ns: *p* > 0.05; * : *p* <  = 0.05; ** : *p* <  = 0.01; ***: *p* <  = 0.001; **** : *p* <  = 0.0001

Based on the combinations of alleles of three markers, four different haplotypes were constructed for *QYr.nmbu.6A*. However, as only three lines in the GWAS panel contain the “G_T_T” haplotype, this haplotype was excluded from the pair-wise comparison due to the lack of statistical power. According to Fig. 2, the first haplotype “A_C_T” showed the highest disease severity among all haplotype groups, while the third haplotype “G_T_G”, which carried alternative alleles of all three markers in comparison to the “A_C_T” haplotype, showed the lowest disease severity. Significant differences of disease severities were consistently detected between these two haplotypes in all 17 tested trials, including those trials where the *QYr.nmbu.6A* was not significantly detected by association analysis. In addition, we observed that the second haplotype “G_C_T”, which only carries an alternative allele of the first marker in comparison to the first susceptible haplotype “A_C_T”, showed medium susceptibility. Significant differences in disease severities between this susceptible haplotype “G_C_T” and resistant haplotype “G_T_G” were also consistently detected in 14 out of the 17 tested trials. Furthermore, in 10 out of the 17 trials, significant differences of disease severity were also detected between the first two susceptible haplotypes. Besides, as shown by Fig. 2, the predominant haplotype in our GWAS panel was “G_C_T” (66.3%), while only 20% of the lines carried the resistant haplotype “G_T_G”, most of which were new varieties and breeding lines from Norway and Sweden.

**Fig. 3:**
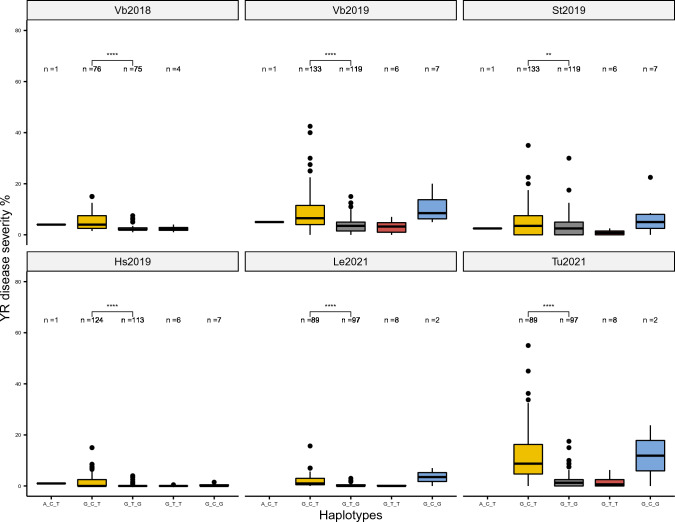
Haplotype analysis of QTL *QYr.nmbu.6A* on Graminor validation panel of trials from six environments. Vb: Vollebekk, Norway; St: Staur, Norway; Hs: Holmestrand, Norway; Le: Lemgo, Germany; Tu: Tulln, Austria; Differences in yellow rust severity (%) between haplotypes were determined by the Wilcoxon test, ns: *p* > 0.05; *: *p* <  = 0.05; **: *p* <  = 0.01; ***: *p* <  = 0.001; ****: *p* <  = 0.0001

To verify the QTL effect of *QYr.nmbu.6A*, the same haplotype analysis was also carried out using the validation panel (Fig. 3). A fifth haplotype “G_C_G” was found in the validation panel. However, as too few lines belong to the first haplotype “A_C_T” (*n* = 1), third haplotype “G_T_T” ( *n*= 8), and the fifth haplotype “G_C_G” (*n*=7), these three haplotypes were excluded from the pair-wise comparisons due to the lack of statistical power. Significant haplotype effects were still consistently identified in all trials between the medium susceptible haplotype “G_C_T” and the resistant haplotype “G_T_G”. Interestingly, the frequency of the resistance haplotype “G_T_G” was much higher in the validation panel (up to 50%), which consisted of new Norwegian breeding lines (Fig. 3).

## Analysis of QTL physical intervals using the Chinese Spring reference genome

As the marker *GENE-4021_496* was the most significant and consistent for the 6A QTL, we also searched for candidate genes around ± 1 Mbp of the marker physical map location. In total, 58 predicted gene models were detected in the region and eighteen gene models were annotated as encoding disease resistance related proteins (bold in Table S4). Among those, eleven gene models were predicted to encode either nucleotide-binding site (NBS) and/or leucine-rich repeats (LRR).

## Stacking resistant alleles

**Fig. 4:**
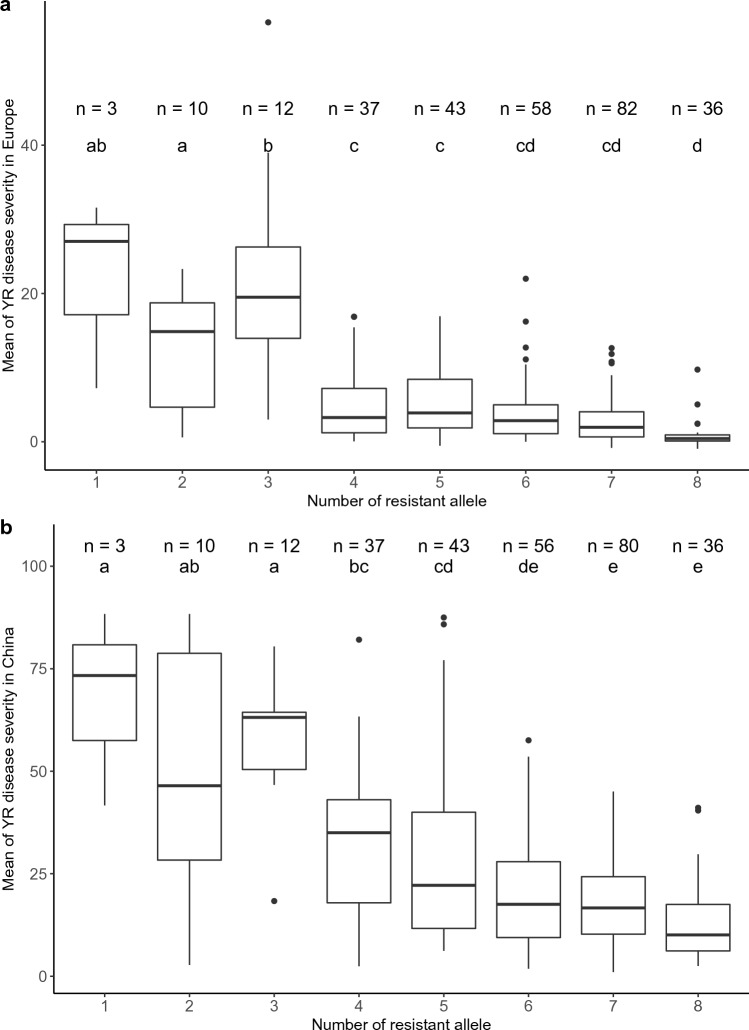
Stacking resistant alleles of the eight QTL which were commonly detected from trials across continents. Different letters on boxplots indicate significant differences (*p* < 0.05) in (**a**: mean of YR disease severity in Europe; ** b**: mean of YR disease severity in China) between groups by Tukey′s HSD test

By accumulating resistant alleles, a descending trend can be observed for mean disease severities of trials in Europe and China (Fig. 4). However, the group of lines which carried two resistant alleles showed relatively lower disease severity in comparison to the third group which carried three resistant alleles for trials in Europe (Fig. 4a), while there were no significant differences of mean disease severities in China among first three groups which carried one, two and three resistant alleles (Fig. 4b). A minimum of two more resistant alleles between groups were needed to obtain statistically significant differences in mean of disease severities.

## Development of KASP markers for *QYr.nmbu.6A* and tracing back the origin of the resistant source

Markers *AX-158600183*, *GENE-4021_496* and *AX-95182345* were used for constructing the haplotype analysis for GWAS and validation panel. However, KASP genotyping of marker *GENE-4021_496* (610 Mbp) was not successful. Therefore, we replaced it by marker *AX-158587919* (609 Mbp), which was in high LD with *GENE-4021_496* in our GWAS panel (*R*^2^ = 0.85). By comparing the alleles of marker *GENE-4021_496* and *AX-158587919* in the GWAS panel, we found that allele “C” of marker *GENE-4021_496* was equivalent to allele “A” of marker *AX-158587919*, while allele “T” of marker *GENE-4021_496* was equivalent to allele “G” of marker *AX-158587919.* When genotyping the old variety panel with KASP markers for *AX-158600183*, *AX-158587919* and *AX-95182345*, 7.9% of lines belonged to the first susceptible haplotype, 43.0% lines were grouped to the second susceptible haplotype, while 10.5% of the lines contained the resistant haplotype. 14.0% of the lines belonged to another haplotype group “G_G_T”, which would be equivalent to the minor haplotype group “G_T_T” in the GWAS panel. The remaining lines contained at least one heterozygous allele or missing value in the KASP genotyping data.

## Discussion

In recent years the predominant yellow rust race group in Europe has been the *PstS10*, which was detected for the first time in Europe in 2012 and the epidemic has continued since 2014 (Ali et al. 2017; Hovmøller et al. 2022). Common adult plant resistance QTL detected by this study, which were specific to European trials, would be useful resources for wheat breeding against this dominant race group. However, as all the detected QTL showed field resistance, they could potentially be useful in providing broad-spectrum resistance. This could also be true for the QTL that were identified only from trials in one continent when being co-located to previously detected QTL showing broad-spectrum resistance. *QYr.nmbu.3A.2* located at 479–484 Mbp on chromosome 3A might be in common with another APR QTL *QYr.spa-3A.1* (480 Mbp) (Bokore et al. 2021), which was detected by field trials conducted in Mexico. *QYr.nmbu.3B* was identified repetitively in Norway and by one trial in Austria on chromosome 3B (818–819 Mbp). We found another APR QTL *QYr.nwafu-3BL.2,* reported by Wu et al. (2020b), which was mapped to an approximately similar location at 793 Mbp on 3B. In addition, *QYr.nmbu.6B* located at the short am of chromosome 6B (94–113 Mbp) was very close to the APR gene *Yr78* (92.4 Mbp) (Dong et al. 2017). Moreover, Tehseen et al. (2020) carried out a GWAS analysis using an isolate mixture of *PstS2* and Warrior (*PstS7*) races for adult plant inoculation in fields, of which one of the significant QTL located at 718 Mbp on 7A quite close to our *QYr.nmbu.7A* (724–731 Mbp). Yao et al. (2020) identified an APR QTL at 54 Mbp by a GWAS analysis using artificial inoculation of a mixture of Chinese yellow rust races, which mapped close to our APR QTL *QYr.nmbu.7B* (62 Mbp). To the best of our knowledge, *QYr.nmbu.2A.1, QYr.nmbu.3D* and *QYr.nmbu.5A.1* do not correspond to any of the previously detected APR QTL for yellow rust and can be considered novel.

*QYr.nmbu.2B.1* and *QYr.nmbu.3A.1* are two QTL which were identified specific to environments in China. In the GWAS study by Wu et al. (2020b), *QYr.nwafu-2BL.2* located at 708 Mbp on 2B, was identified by field experiments in China, while the QTL interval of *QYr.nmbu.2B.1* was between 711 to 722 Mbp, which could be a candidate QTL for improving yellow rust resistance in China. Furthermore, although *QYr.nmbu.3A.1* (7 Mbp) was identified only by trials conducted in China, quite a few published APR QTL have been reported in this short arm region of chromosome 3A (Rosewarne et al. 2013; Yao et al. 2020; Bouvet et al. 2022b). As the QTL was reported by field studies carried out in different continents using different mapping germplasms, it illustrated that the QTL might be a durable and potentially race non-specific QTL.

The main focus of our study was to identify APR yellow rust resistance QTL which could be suitable for broad deployment. As mentioned in the previous section, the dominant yellow rust races in Europe belong to the *PstS10* group, however, they are likely different from the dominant races in China, Kenya and Mexico (Ali et al. 2017; Hovmøller et al. 2022). Thus, the QTL we identified across continents implied broad resistance against different yellow rust race groups. Moreover, if the APR QTL were also published by other studies either using different mapping materials or disease trials with different races, it provides further evidence for potential broad-spectrum resistance. For example, *QYr.nmbu.1B* was detected by trials in Germany and China in our study. Through literature review, we found an APR QTL reported by Tehseen et al. (2020) which mapped to a region about 10 Mbp apart from the interval of *QYr.nmbu.1B. QYr.nmbu.2A.2* (717–729 Mbp) was commonly detected by trials conducted in China and Kenya, which was mapped close to an APR QTL (717 Mbp) reported by Tehseen et al. (2020) and *QYr.cim-2AL* (719.5–728.8 Mbp) by Yuan et al. (2020). We also detected a common QTL between China and Kenya on 2D which was caused by the photoperiod gene *PpD-D1* (Beales et al. 2007). Although it explained up to 15% of the phenotypic variation, this was likely an artifact due to the variations in photoperiod responses within the AM panel and different photoperiods in Kenya and China in comparison to trials conducted in central and northern Europe. *QYr.nmbu.5B.1* was another QTL that we detected across trials from Europe and China. Although not located at the QTL interval of *QYr.nmbu.5B.1* (515–518 Mbp), Tehseen et al. (2020) found an APR QTL mapped to 507 Mbp on chromosome 5B. In addition, a seedling resistance QTL *QYr.nwafu-5BL* reported by Wu et al. (2020a) was also mapped close to *QYr.nmbu.5B.1* at 523 Mbp. *QYr.nmbu.4A*, *QYr.nmbu.4D, QYr.nmbu.5A.2* and *QYr.nmbu.5B.2* are likely novel APR QTL reported for the first time by our study.

Stacking multiple QTL for yellow rust resistance in wheat has been the approach to enhance durable resistance, given the limited long-term effectiveness of single ASR genes. Despite the practical challenges such as limited knowledge of the APR genes, the time cost and linkage drags of unwanted agronomic traits, pyramiding APR genes may offer greater durability for yellow rust resistance. Notably, Wang et al. (2023) demonstrated that stacking yellow rust APR genes *Yr18*, *Yr28* and *Yr36* could increase both field and seedling resistances. Our study also provided evidence that the combination of resistant QTL alleles detected across continents can reduce disease severities in both Europe and China. However, continued research efforts are necessary to ensure the long-term effectiveness of stacked QTL. The application of gene editing techniques may offer a promising approach to stacking multiple alleles and circumventing the limitations of traditional breeding methods.

The last but not the least, *QYr.nmbu.6A* was the most consistent and significant QTL in this study. It showed up in 9 out of the 17 tested trials by GWAS, and the haplotype effects were consistently detected across all environments, although the QTL was not detected by GWAS from trials conducted in Germany, Kenya, and Mexico. As we described in the materials and methods, we observed a shift of *QYr.nmbu.6A* interval from 598–610 Mbp to 610–612 Mbp when changing from original to transformed phenotypic dataset. However, the marker *GENE-4021_496* was always significant regardless of the data transformation. In addition, through LD analysis, we found the marker *AX-95182345* (612 Mbp) to be in high LD (*R*^2^ = 0.94) with the most consistent marker *GENE-4021_496* (610 Mbp). According to the haplotype analysis, we found significant differences between the susceptible haplotypes “A_C_T” and “G_C_T” in ten out of the seventeen tested trials. Based on this we could expand the QTL interval of *QYr.nmbu.6A* to 598–612 Mbp. We have also noticed that quite a few studies which published recently also reported yellow rust APR QTL at the same region on chromosome 6A. Bouvet et al. (2022b) detected a minor APR QTL *QYr.niab-6A.3* at 596.5 Mbp on 6A by QTL mapping of the NIAB MAGIC population. While Beukert et al. (2020) conducted a GWAS analysis and mapped an APR QTL associated to marker *Tdurum_contig29607_413* at 609.4 Mbp and marker *GENE-4021_496* at 610 Mbp. The *GENE-4021_496* was the same significant marker as what we identified for *QYr.nmbu.6A*. Another GWAS by Shahinnia et al. (2022) also detected the significant marker *Tdurum_contig29607_413* in their yellow APR resistance study. Moreover, Kale et al. (2022) mapped a yellow rust APR QTL within the 611.98 to 612.66 Mbp interval on 6A of the ‘Attraktion’ genome. For comparison, we obtained the sequences and anchored the interval to 610.6 to 610.9 Mbp on Chinese spring IWGSC RefSeq v1.0 (International Wheat Genome Sequencing et al. 2018).

Interestingly, the study of Kale et al. (2022) compared the haplotype of the targeted 6A region of the ′Attraktion′ genome with the wheat pan genomes, by which they discovered that only ′SY Mattis′ carried the same haplotype as ′Attraktion′. Through marker sequence comparisons with the wheat pan genomes and ′Attraktion′ genome (Walkowiak et al. 2020; Kale et al. 2022), we confirmed that ′SY Mattis′ harbored the “G_T_T” haplotype and ′Attraktion′ harbored the “G_T_G” haplotype, while the other wheat cultivars in the pan-genome project carried either of the two susceptible haplotypes in our haplotype analysis. It revealed that the 6A resistance QTL from our study presumably was the same as reported by Kale et al. (2022). As mentioned previously, the resistant haplotype is more common in the new cultivars and breeding lines than the old varieties. ′Alve′, the Swedish cultivar which was released in 1992 was the oldest line included in our GWAS panel which contained this resistant haplotype, and it was the resistance source of some recent Norwegian cultivars. Therefore, we traced back the pedigree of ′Avle′ and KASP genotyped a panel of historical Nordic spring wheat varieties and landraces. Based on our KASP genotyping results, only 12 out of the 114 genotyped lines carried the resistant haplotype, in addition to the positive control line ′Avle′. Most historical lines with the resistant haplotype are of Swedish origin except one Norwegian landrace which to our knowledge was not deployed in wheat breeding in Norway. ′Atle′ (1936), ′Blanka′ (1950) and ′Atson′ (1954) are the oldest cultivars in the old variety panel containing the resistant haplotype. All of them have the British winter wheat cultivar ′Squarehead′ (1830) in their pedigree, which is the most likely the source of resistance, since the other two common cultivars in the pedigrees, ′Extra-kolben I′ and ′Extra-kolben II′, carried the susceptible haplotype. In accordance with our haplotype analysis and KASP genotyping results, Kale et al. (2022) also found low frequency of the resistant haplotype in landraces and varieties before 1970, which illustrates that the frequency of the resistance haplotype might have accumulated in breeding materials recently through selection and may also link to the shift of the dominant yellow rust races in Europe (Ali et al. 2017).

Analysis of the ± 1 Mbp interval of the significant marker *GENE-4021_496* identified eighteen annotated disease resistance genes, of which eleven encodes NBS and/or LRR domains (Table S4). However, as reviewed by Hafeez et al. (2021), none of the APR genes being cloned so far encode NLR. Since the *QYr.nmbu.6A* was presumed to be caused by an APR gene, the candidate gene prediction result is not conclusive as further validation at seedling stage and confirmation of the corresponding yellow rust races are required. As far as we know, this 6A QTL was only published in previous studies based on European winter wheat materials assessed in field trials conducted in Europe. Our study reported this 6A QTL for the first time in spring wheat material, and also validated the QTL effect in other continents where the dominant yellow rust races are likely different from Europe. As the resistant haplotype is frequently available in new elite cultivars, broad-spectrum APR resistance can be achieved by marker assisted selection within the elite gene pool.

## Supplementary Information

Below is the link to the electronic supplementary material.

Supplementary file1 (PDF 10 kb) Fig. S1 Histogram of the original yellow rust disease severity data (%) from seventeen field trials of the NMBUspring wheat panel. Vb: Vollebekk, Norway; St: Staur, Norway; Hs: Holmestrand, Norway; FK: Feldkirchen,Germany; Tu: Tulln, Austria; XD: Xindu, China; PX: Pixian, China; Keny: Kenya; Tolu: Toluca, Mexico

Supplementary file2 (PDF 15 kb) Fig. S2 Pairwise comparison in mean of YR disease severity in Europe between two alleles of the ninesignificant MTAs used for allele stacking by Wilcoxon test. ns: p > 0.05; *: p <= 0.05; **: p <= 0.01; ***: p <=0.001; ****: p <= 0.0001

Supplementary file3 (PDF 11 kb) Fig. S3 Pairwise comparison in mean of YR disease severity in China between two alleles of the ninesignificant MTAs used for allele stacking by Wilcoxon test. ns: p > 0.05; *: p <= 0.05; **: p <= 0.01; ***: p <=0.001; ****: p <= 0.0001

Supplementary file4 (PDF 10 kb) Fig. S4 Histogram of the transformed yellow rust disease severity data from seventeen field trials of the NMBUspring wheat panel. Vb: Vollebekk, Norway; St: Staur, Norway; Hs: Holmestrand, Norway; FK: Feldkirchen,Germany; Tu: Tulln, Austria; XD: Xindu, China; PX: Pixian, China; Keny: Kenya; Tolu: Toluca, Mexico

Supplementary file5 (PDF 5282 kb) Fig. S5 QQ plots of marker-trait association for adult plant yellow rust disease severity in the NMBU springwheat panel. Vb: Vollebekk, Norway; St: Staur, Norway; Hs: Holmestrand, Norway; FK: Feldkirchen,Germany; Tu: Tulln, Austria; XD: Xindu, China; PX: Pixian, China; Keny: Kenya; Tolu: Toluca, Mexico.Markers from analysis using the original phenotypic data (Ori) were indicated in red dots, while markers from analysis using the transformed phenotypic data (Trans) were indicated in blue dots

Supplementary file6 (PNG 341 kb) Fig. S6 Manhattan plots of the adult plant yellow rust disease severity in the NMBU spring wheat panel. Vb:Vollebekk, Norway; St: Staur, Norway; Hs: Holmestrand, Norway; FK: Feldkirchen, Germany; Tu: Tulln, Austria;XD: Xindu, China; PX: Pixian, China; Keny: Kenya; Tolu: Toluca, Mexico. The significance threshold set toFDR adjusted p-value below 0.05. Markers above the significant threshold are indicated in red dots

Supplementary file7 (XLSX 31 kb) Table S1 List of 3 KASP markers developed in this study for tracing the origin of resistant allele. Table S4 Overview of candidate genes of QTL *QYr.nmbu.6A*, physical positions were based on the wheat reference genome (RefSeq v1.0), and gene annotations was based on HighConfidenceGenesv1.1. Table S5 KASP genotyping results of the old cultivar panel

Supplementary file8 (DOCX 43 kb) Table S2 Markers used for allele stacking and mean of disease severity for each allele. Table S3 Significant markers associated with yellow rust disease severity in the NMBU spring wheat panel tested in seventeen trials, at seven locations from 2015 to 2021. Vb: Vollebekk, Norway; St: Staur, Norway; Hs: Holmestrand, Norway; FK: Feldkirchen, Germany; Tu: Tull, Austria; XD: Xindu, China; PX: Pixian, China, Keny: Kenya; Tulo: Toluca, Mexico. Physical positions were based on blastn result against reference genome IWGSC RefSeq v1.0 (International Wheat Genome Sequencing et al. 2018)

Supplementary file9 (CSV 17 kb)

## Data Availability

The population and genotype data used in current study were described by Lin et al. (2022). Phenotypic data generated during this study are included in this published article and it′s supplementary information files.
