# Supplementary figures and images for: A major yellow rust resistance QTL on chromosome 6A shows increased frequency in recent Norwegian spring wheat cultivars and breeding lines
Source: Theor Appl Genet. 2023 Jul 1;136(7):164. doi: 10.1007/s00122-023-04397-9 (PMC10314843; doi:10.1007/s00122-023-04397-9)

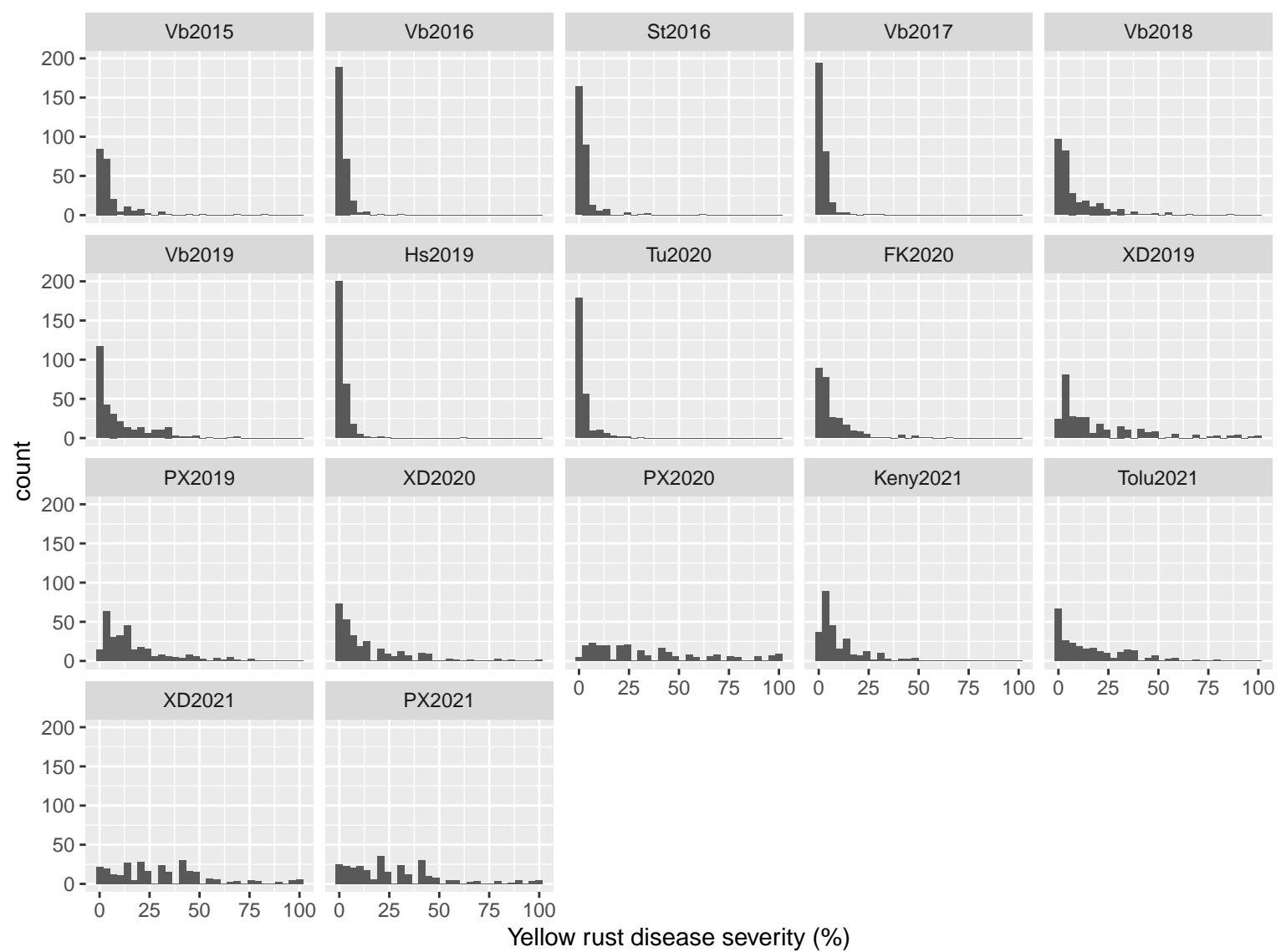

Supplement: Supplementary file 1 — Supplementary file1 (PDF 10 kb) Fig. S1 Histogram of the original yellow rust disease severity data (%) from seventeen field trials of the NMBUspring wheat panel. Vb: Vollebekk, Norway; St: Staur, Norway; Hs: Holmestrand, Norway; FK: Feldkirchen,Germany; Tu: Tulln, Austria; XD: Xindu, China; PX: Pixian, China; Keny: Kenya; Tolu: Toluca, Mexico [file 122_2023_4397_MOESM1_ESM.pdf]

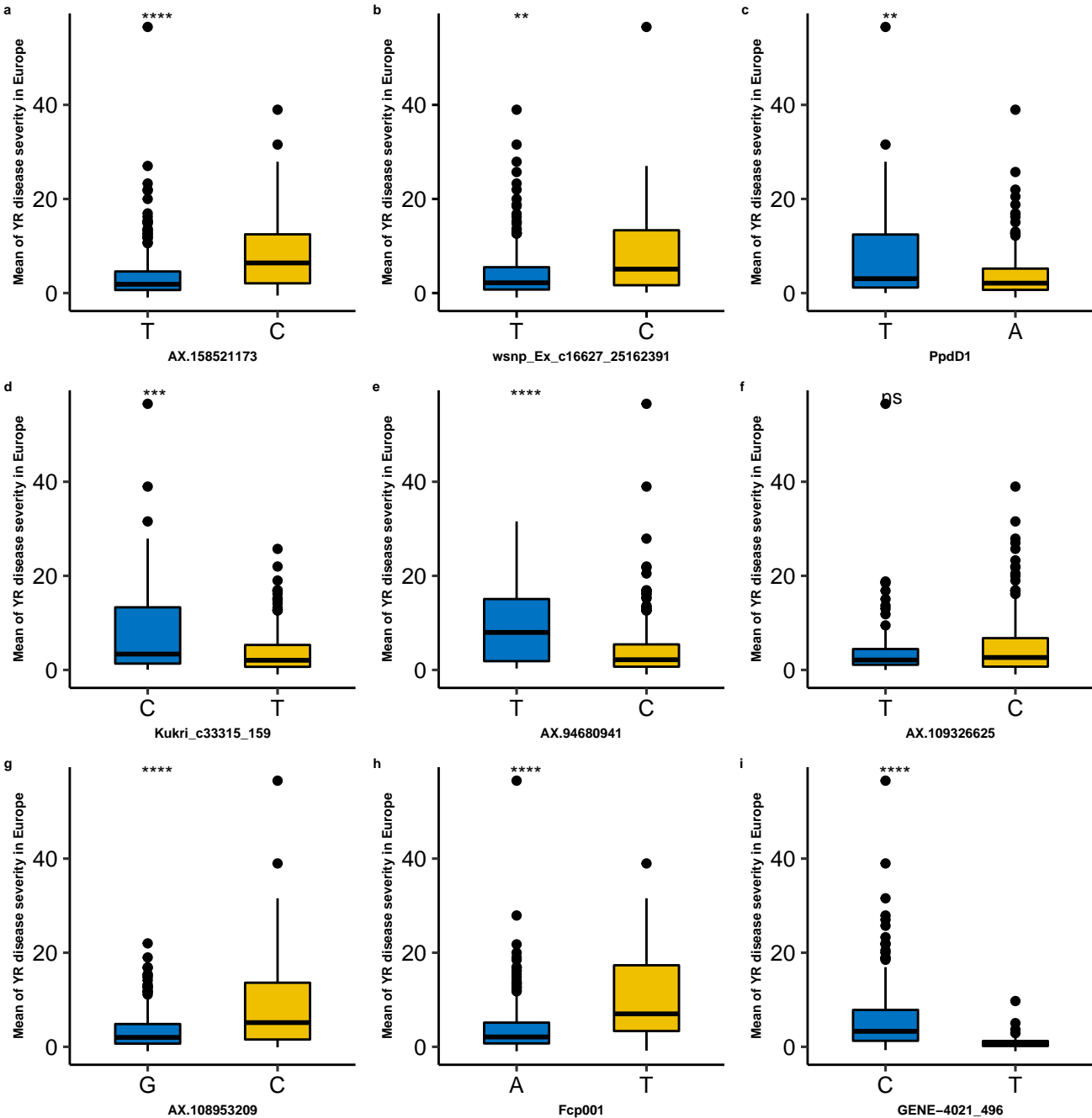

Supplement: Supplementary file 2 — Supplementary file2 (PDF 15 kb) Fig. S2 Pairwise comparison in mean of YR disease severity in Europe between two alleles of the ninesignificant MTAs used for allele stacking by Wilcoxon test. ns: p > 0.05; *: p <= 0.05; **: p <= 0.01; ***: p <=0.001; ****: p <= 0.0001 [file 122_2023_4397_MOESM2_ESM.pdf]

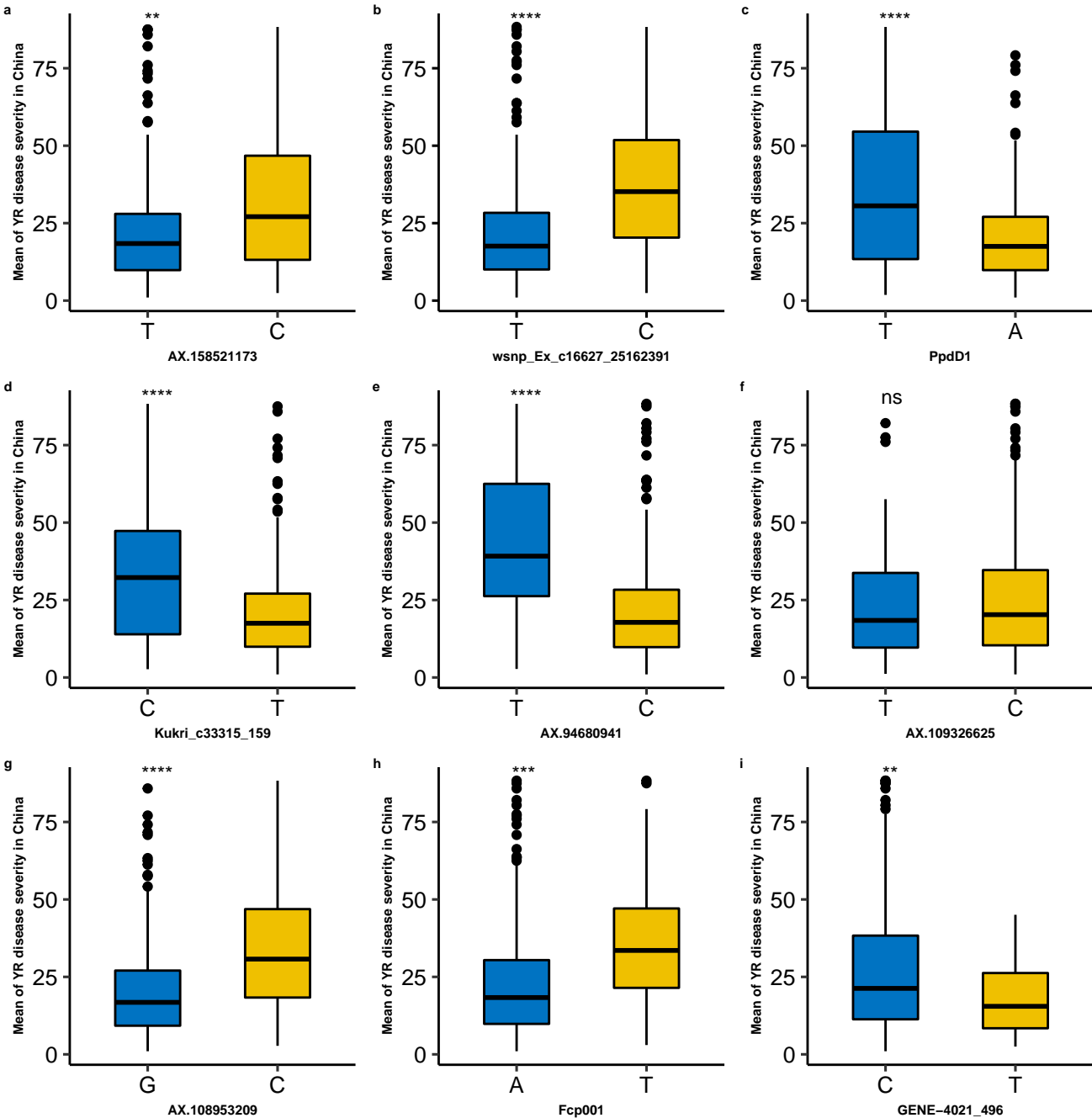

Supplement: Supplementary file 3 — Supplementary file3 (PDF 11 kb) Fig. S3 Pairwise comparison in mean of YR disease severity in China between two alleles of the ninesignificant MTAs used for allele stacking by Wilcoxon test. ns: p > 0.05; *: p <= 0.05; **: p <= 0.01; ***: p <=0.001; ****: p <= 0.0001 [file 122_2023_4397_MOESM3_ESM.pdf]

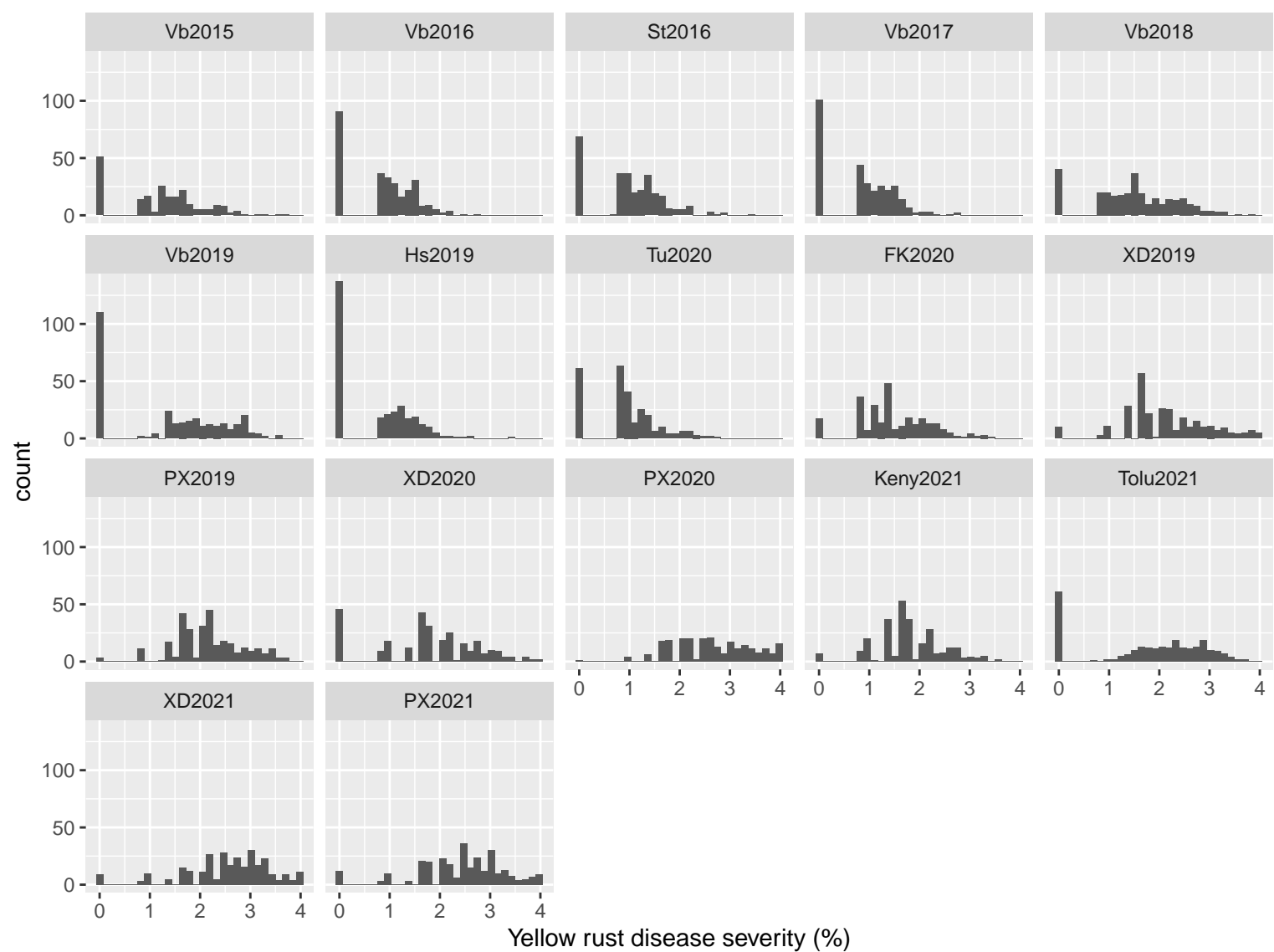

Supplement: Supplementary file 4 — Supplementary file4 (PDF 10 kb) Fig. S4 Histogram of the transformed yellow rust disease severity data from seventeen field trials of the NMBUspring wheat panel. Vb: Vollebekk, Norway; St: Staur, Norway; Hs: Holmestrand, Norway; FK: Feldkirchen,Germany; Tu: Tulln, Austria; XD: Xindu, China; PX: Pixian, China; Keny: Kenya; Tolu: Toluca, Mexico [file 122_2023_4397_MOESM4_ESM.pdf]

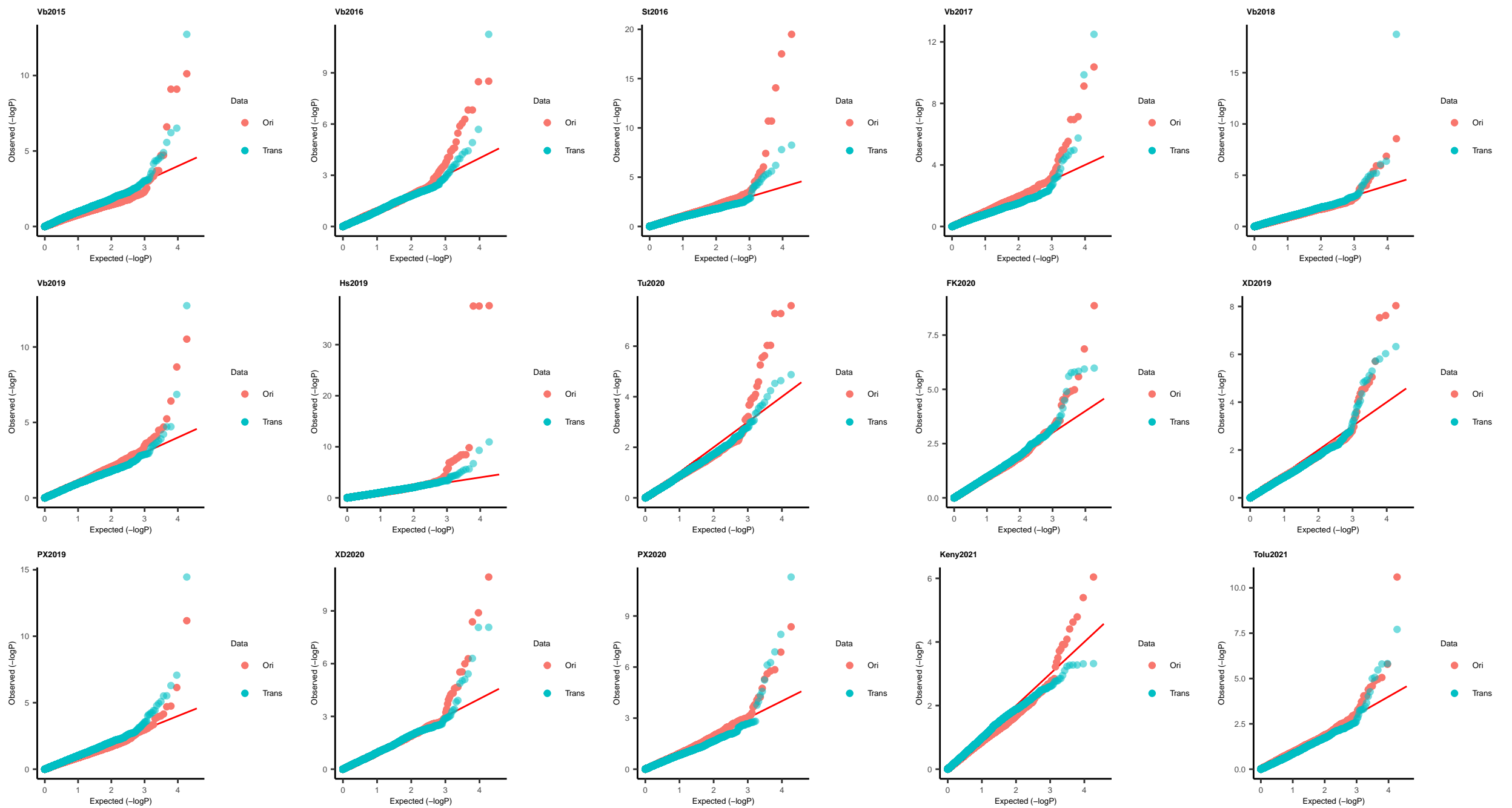

Supplement: Supplementary file 5 — Supplementary file5 (PDF 5282 kb) Fig. S5 QQ plots of marker-trait association for adult plant yellow rust disease severity in the NMBU springwheat panel. Vb: Vollebekk, Norway; St: Staur, Norway; Hs: Holmestrand, Norway; FK: Feldkirchen,Germany; Tu: Tulln, Austria; XD: Xindu, China; PX: Pixian, China; Keny: Kenya; Tolu: Toluca, Mexico.Markers from analysis using the original phenotypic data (Ori) were indicated in red dots, while markers from analysis using the transformed phenotypic data (Trans) were indicated in blue dots [file 122_2023_4397_MOESM5_ESM.pdf]

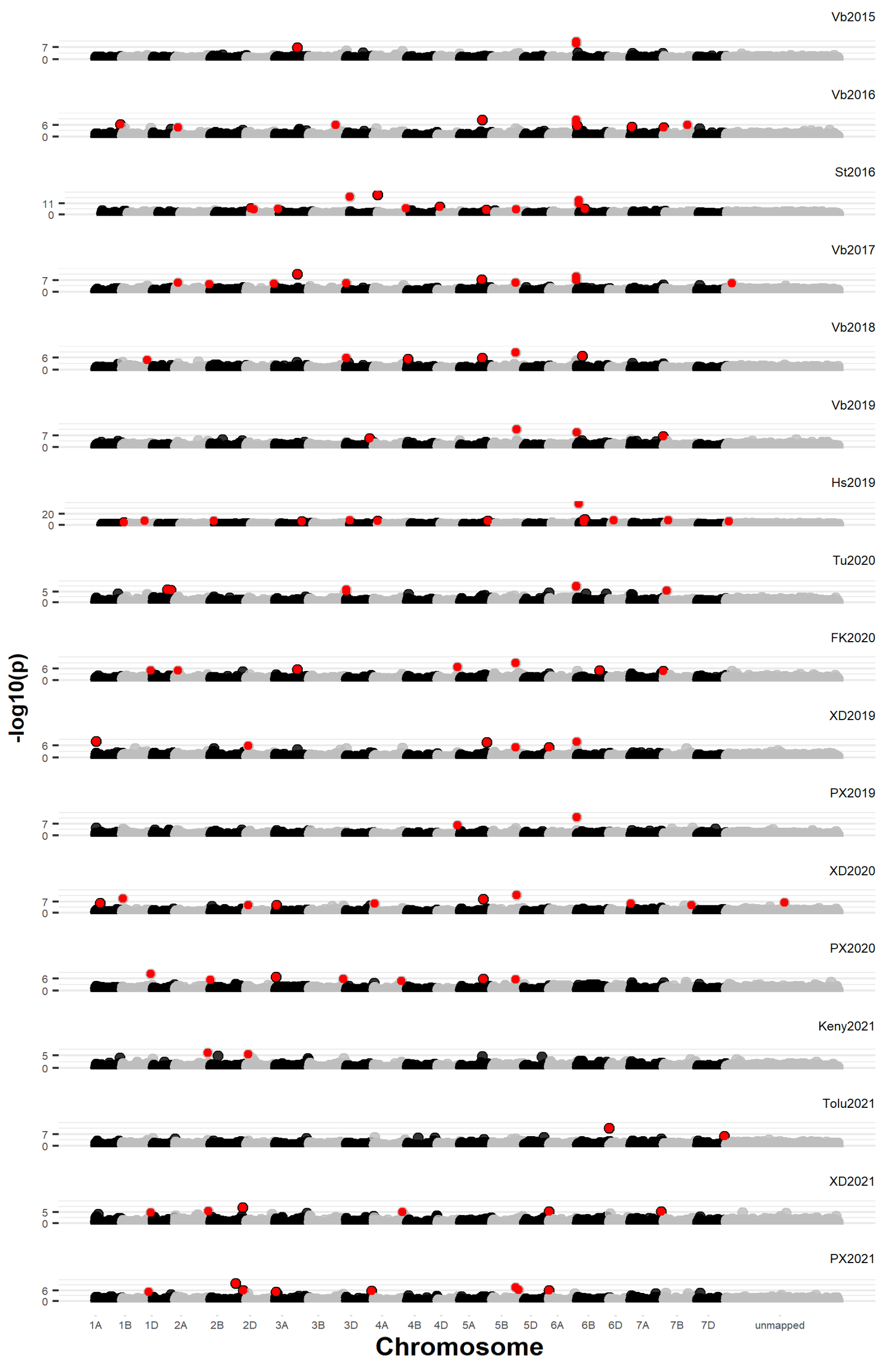

Supplement: Supplementary file 6 — Supplementary file6 (PNG 341 kb) Fig. S6 Manhattan plots of the adult plant yellow rust disease severity in the NMBU spring wheat panel. Vb:Vollebekk, Norway; St: Staur, Norway; Hs: Holmestrand, Norway; FK: Feldkirchen, Germany; Tu: Tulln, Austria;XD: Xindu, China; PX: Pixian, China; Keny: Kenya; Tolu: Toluca, Mexico. The significance threshold set toFDR adjusted p-value below 0.05. Markers above the significant threshold are indicated in red dots [file 122_2023_4397_MOESM6_ESM.png]
