# Supplementary material for: A major yellow rust resistance QTL on chromosome 6A shows increased frequency in recent Norwegian spring wheat cultivars and breeding lines
Source: Theor Appl Genet. 2023 Jul 1;136(7):164. doi: 10.1007/s00122-023-04397-9 (PMC10314843; doi:10.1007/s00122-023-04397-9)
Supplement: Supplementary file 8 — Supplementary file8 (DOCX 43 kb) Table S2 Markers used for allele stacking and mean of disease severity for each allele. Table S3 Significant markers associated with yellow rust disease severity in the NMBU spring wheat panel tested in seventeen trials, at seven locations from 2015 to 2021. Vb: Vollebekk, Norway; St: Staur, Norway; Hs: Holmestrand, Norway; FK: Feldkirchen, Germany; Tu: Tull, Austria; XD: Xindu, China; PX: Pixian, China, Keny: Kenya; Tulo: Toluca, Mexico. Physical positions were based on blastn result against reference genome IWGSC RefSeq v1.0 (International Wheat Genome Sequencing et al. 2018) [file 122_2023_4397_MOESM8_ESM.docx]

Table S2: Markers used for allele stacking and mean of disease severity for each allele

| Marker | Chromosome | Physical position (Mbp) | Alleles (Resistant/ Susceptible) | Mean of YR disease severity from trials in Europe for each allele (%) | Mean of YR disease severity from trials in China for each allele (%) |
| --- | --- | --- | --- | --- | --- |
| AX-158521173 | 1B | 629 | T/A | 3.91/8.30 | 22.42/32.88 |
| wsnp_Ex_c16627_25162391 | 2A | 717 | T/A | 4.27/8.39 | 22.61/36.51 |
| PpdDD001 | 2D | 34 | A/T | 3.97/7.29 | 20.45/35.93 |
| Kukri_c33315_159 | 4A | 631 | T/C | 3.79/8.69 | 21.57/35.62 |
| AX-94680941 | 4D | 439 | C/T | 4.25/10.15 | 23.66/25.33 |
| AX-108953209 | 5B | 515 | G/C | 3.56/9.03 | 20.98/36.34 |
| AX-109326625 | 5A | 503 | T/C | 4.64/5.04 | 23.66/25.33 |
| fcp001 | 5B | 546 | A/T | 4.15/11.05 | 23.35/36.87 |
| GENE-4021_496 | 6A | 610 | T/C | 0.97/6.04 | 17.32/27.06 |

Table S3 Significant markers associated with yellow rust disease severity in the NMBU spring wheat panel tested in seventeen trials, at seven locations from 2015 to 2021. Vb: Vollebekk, Norway; St: Staur, Norway; Hs: Holmestrand, Norway; Le: Lemgo, Germany; Tu: Tull, Austria; XD: Xindu, China; PX: Pixian, China, Keny: Kenya; Tulo: Toluca, Mexico. Physical positions were based on blastn result against reference genome IWGSC RefSeq v1.0 (International Wheat Genome Sequencing et al. 2018)

| Trial | Chromosome | SNP | Physical position (Mbp) | FDR Adjusted  P values | effect | -log10(p) |
| --- | --- | --- | --- | --- | --- | --- |
| Vb 2015 | 3A | AX-94458766 | 479 | 1 .16E-03 | 5 .80 | 6 .60 |
|  | 6A | AX-158600183 | 598 | 4 .97E-06 | 52 .45 | 9 .10 |
|  | 6A | RFL_Contig6053_2072 | 598 | 1 .41E-06 | 2 .26 | 10 .12 |
|  | 6A | RFL_Contig6053_3082 | 598 | 4 .97E-06 | -26 .23 | 9 .10 |
| Vb 2016 | 1A | AX-94408000 | 550 | 1 .95E-03 | -0 .63 | 6 .28 |
|  | 2A | AX-94639471 | 52 | 4 .56E-02 | -0 .64 | 4 .60 |
|  | 3B | Kukri_c5347_811 | 591 | 3 .48E-03 | -0 .64 | 5 .88 |
|  | 5A | Tdurum_contig59338_1902 | 489 | 3 .08E-05 | -1 .63 | 8 .51 |
|  | 6A | AX-158600183 | 598 | 6 .97E-04 | 13 .45 | 6 .82 |
|  | 6A | RFL_Contig6053_2072 | 598 | 3 .08E-05 | 0 .98 | 8 .48 |
|  | 6A | RFL_Contig6053_3082 | 598 | 6 .97E-04 | -6 .72 | 6 .82 |
|  | 6B | Kukri_c21405_1705 | 1 | 7 .94E-03 | -1 .23 | 5 .47 |
|  | 7A | AX-109323587 | 20 | 2 .30E-02 | -1 .12 | 4 .95 |
|  | 7A | RFL_Contig2814_604 | 731 | 4 .56E-02 | 0 .87 | 4 .57 |
|  | 7B | wsnp_BE443010B_Ta_2_2 | 519 | 2 .77E-03 | 0 .95 | 6 .05 |
| Vb 2017 | 2A | AX-95242673 | 58 | 1 .27E-02 | 0 .91 | 5 .32 |
|  | 2A | AX-158540732 | 759 | 4 .56E-02 | 0 .71 | 4 .57 |
|  | 2D | AX-95085281 | 619 | 4 .07E-02 | 0 .74 | 4 .66 |
|  | 3A | wsnp_JD_c5699_6859527 | 484 | 7 .92E-07 | -1 .68 | 10 .37 |
|  | 3B | AX-158558043 | 819 | 2 .17E-02 | 0 .70 | 5 .00 |
|  | 5A | AX-158565245 | 470 | 4 .15E-04 | 1 .03 | 7 .14 |
|  | 5B | AX-108953209 | 515 | 9 .03E-03 | -0 .92 | 5 .54 |
|  | 6A | AX-158600183 | 598 | 4 .15E-04 | 15 .10 | 6 .95 |
|  | 6A | RFL_Contig6053_2072 | 598 | 6 .93E-06 | 1 .41 | 9 .13 |
|  | 6A | RFL_Contig6053_3082 | 598 | 4 .15E-04 | -7 .55 | 6 .95 |
|  | UN | AX-95175956 |  | 2 .17E-02 | 0 .97 | 4 .98 |
| Vb 2018 | 1B | IACX9308 | 551 | 4 .48E-02 | -3 .97 | 4 .84 |
|  | 3B | AX-158558043 | 819 | 5 .64E-03 | 2 .89 | 5 .95 |
|  | 4B | AX-110907280 | 13 | 1 .54E-02 | -2 .11 | 5 .38 |
|  | 5A | Tdurum_contig59338_1902 | 489 | 5 .64E-03 | -4 .61 | 5 .92 |
|  | 5B | AX-108953209 | 515 | 5 .07E-05 | -4 .07 | 8 .56 |
|  | 6B | Tdurum_contig43872_549 | 113 | 1 .29E-03 | -2 .97 | 6 .86 |
| Vb 2019 | 3D | AX-94603646 | 516 | 2 .69E-02 | 2 .95 | 5 .24 |
|  | 5B | fcp001_357 | 546 | 5 .62E-07 | 5 .71 | 10 .52 |
|  | 6A | GENE-4021_496 | 610 | 1 .97E-05 | -3 .96 | 8 .67 |
|  | 7A | Kukri_c18148_913 | 724 | 2 .38E-03 | 2 .70 | 6 .42 |
| St 2016 | 2B | AX-158562114 | 793 | 2 .40E-03 | -1 .70 | 6 .04 |
|  | 2D | wsnp_Ex_c6400_11123059 | 63 | 3 .63E-02 | -1 .30 | 4 .63 |
|  | 2D | wsnp_RFL_Contig1892_1042675 | 602 | 6 .72E-03 | -2 .19 | 5 .49 |
|  | 3B | AX-94390743 | 818 | 2 .89E-14 | 4 .59 | 17 .51 |
|  | 3D | AX-94580748 | 610 | 5 .97E-16 | -4 .25 | 19 .49 |
|  | 4A | Kukri_c33315_159 | 631 | 5 .57E-03 | -1 .38 | 5 .62 |
|  | 4B | AX-95116929 | 643 | 1 .20E-04 | -1 .89 | 7 .41 |
|  | 5A | TA004548-0753 | 508 | 4 .30E-02 | 1 .07 | 4 .52 |
|  | 5B | wsnp_Ex_c974_1864971 | 473 | 3 .03E-02 | -1 .96 | 4 .75 |
|  | 6A | AX-158600183 | 598 | 7 .53E-08 | 31 .79 | 10 .69 |
|  | 6A | RFL_Contig6053_2072 | 598 | 5 .37E-11 | 3 .01 | 14 .06 |
|  | 6A | RFL_Contig6053_3082 | 598 | 7 .53E-08 | -15 .90 | 10 .69 |
|  | 6B | Tdurum_contig43872_549 | 113 | 1 .41E-02 | -1 .25 | 5 .12 |
| Hs 2019 | 1A | AX-158569260 | 507 | 4 .14E-02 | 1 .84 | 4 .33 |
|  | 1A | wsnp_BM140362A_Ta_2_2 | 507 | 4 .14E-02 | -1 .84 | 4 .33 |
|  | 1B | Kukri_c76762_166 | 374 | 1 .43E-04 | 1 .43 | 6 .94 |
|  | 2A | Ra_c3750_1082 | 744 | 1 .50E-04 | 1 .32 | 6 .89 |
|  | 3A | IAAV488 | 482 | 2 .68E-03 | 1 .93 | 5 .59 |
|  | 3B | AX-94390743 | 818 | 2 .01E-05 | 2 .03 | 8 .06 |
|  | 3D | AX-94580748 | 610 | 9 .48E-05 | -1 .56 | 7 .18 |
|  | 5A | AX-158550796 | 544 | 1 .43E-04 | -1 .35 | 6 .94 |
|  | 6A | AX-158600183 | 598 | 1 .68E-34 | 0 .26 | 37 .64 |
|  | 6A | RFL_Contig6053_2072 | 598 | 1 .68E-34 | 46 .00 | 37 .57 |
|  | 6A | RFL_Contig6053_3082 | 598 | 1 .68E-34 | -46 .00 | 37 .57 |
|  | 6B | BS00022823_51 | 94 | 3 .22E-03 | 3 .33 | 5 .48 |
|  | 6B | Tdurum_contig62803_286 | 94 | 8 .51E-05 | -4 .73 | 7 .26 |
|  | 6B | AX-158552701 | 96 | 3 .26E-05 | 5 .05 | 7 .80 |
|  | 6B | AX-111531424 | 101 | 1 .03E-05 | 6 .23 | 8 .41 |
|  | 6B | AX-158552704 | 104 | 1 .03E-05 | 6 .24 | 8 .44 |
|  | 6B | BobWhite_rep_c64102_331 | 109 | 1 .03E-05 | -6 .24 | 8 .44 |
|  | 6B | Tdurum_contig43872_549 | 113 | 6 .87E-07 | -0 .76 | 9 .83 |
|  | 6D | Kukri_rep_c106450_71 | 42 | 6 .95E-05 | -4 .77 | 7 .39 |
|  | 7B | Kukri_c14766_484 | 62 | 3 .74E-05 | 2 .33 | 7 .70 |
|  | UN | AX-158581227 |  | 1 .67E-03 | 0 .96 | 5 .82 |
| Le 2020 | 1B | Tdurum_contig65853_242 | 629 | 4 .32E-02 | 2 .16 | 4 .85 |
|  | 2A | RAC875_c4015_2175 | 58 | 4 .32E-02 | 1 .94 | 4 .98 |
|  | 3A | wsnp_JD_c5699_6859527 | 484 | 1 .63E-02 | -3 .01 | 5 .58 |
|  | 4D | AX-94680941 | 439 | 1 .28E-03 | 3 .29 | 6 .86 |
|  | 5B | AX-108953209 | 515 | 2 .60E-05 | -3 .29 | 8 .85 |
|  | 6B | wsnp_Ex_c14101_22012676 | 492 | 4 .32E-02 | -1 .93 | 4 .92 |
|  | 7A | Kukri_c18148_913 | 724 | 4 .64E-02 | 2 .01 | 4 .76 |
| Tu 2020 | 1D | AX-95220187 | 316 | 3 .47E-03 | -1 .39 | 6 .04 |
|  | 1D | AX-94679138 | 391 | 7 .35E-03 | 1 .31 | 5 .62 |
|  | 3B | AX-158558043 | 819 | 3 .47E-03 | 0 .68 | 6 .03 |
|  | 3B | AX-158579096 | 819 | 1 .29E-02 | 3 .95 | 5 .26 |
|  | 6A | AX-158600183 | 598 | 3 .17E-04 | 18 .00 | 7 .29 |
|  | 6A | RFL_Contig6053_2072 | 598 | 3 .17E-04 | 0 .43 | 7 .60 |
|  | 6A | RFL_Contig6053_3082 | 598 | 3 .17E-04 | -9 .00 | 7 .29 |
|  | 7B | Kukri_c14766_484 | 62 | 7 .49E-03 | 1 .87 | 5 .55 |
| PX 2019 | 4D | AX-94680941 | 439 | 6 .69E-03 | 5 .43 | 6 .14 |
|  | 6A | GENE-4021_496 | 610 | 1 .26E-07 | -6 .12 | 11 .17 |
| PX 2020 | 1B | AX-158521173 | 629 | 8 .00E-05 | -7 .93 | 8 .37 |
|  | 2A | AX-109507247 | 780 | 1 .58E-02 | 10 .16 | 5 .29 |
|  | 3A | AX-158577637 | 7 | 1 .23E-03 | 6 .50 | 6 .88 |
|  | 3B | AX-94626857 | 760 | 7 .90E-03 | -7 .74 | 5 .77 |
|  | 4A | IAAV7132 | 614 | 4 .72E-02 | 5 .59 | 4 .75 |
|  | 5A | AX-109308419 | 504 | 7 .90E-03 | 6 .41 | 5 .84 |
|  | 5B | AX-108953209 | 515 | 9 .27E-03 | -6 .70 | 5 .60 |
| XD 2019 | 1A | AX-94844830 | 12 | 1 .72E-04 | -10 .05 | 8 .03 |
|  | 2D | PpdDD001 | 34 | 9 .16E-03 | 5 .55 | 5 .71 |
|  | 5A | TA001896-0654 | 589 | 1 .83E-04 | 10 .30 | 7 .53 |
|  | 5B | wsnp_Ku_c14202_22436656 | 518 | 3 .30E-02 | 4 .30 | 5 .05 |
|  | 5D | BS00089081_51 | 550 | 4 .57E-02 | -4 .38 | 4 .83 |
|  | 6A | wsnp_JD_c7795_8868122 | 607 | 1 .83E-04 | 9 .80 | 7 .62 |
| XD 2020 | 1A | AX-94729463 | 102 | 3 .78E-03 | -6 .72 | 5 .99 |
|  | 1B | AX-158607148 | 7 | 1 .19E-05 | 4 .89 | 8 .89 |
|  | 2D | PpdDD001 | 34 | 4 .73E-02 | 3 .92 | 4 .67 |
|  | 3A | AX-158577605 | 20 | 4 .74E-02 | 3 .17 | 4 .59 |
|  | 4A | BS00108852_51 | 11 | 8 .00E-03 | -3 .76 | 5 .52 |
|  | 5A | AX-109326625 | 503 | 2 .59E-05 | -5 .82 | 8 .38 |
|  | 5B | fcp001 | 546 | 2 .20E-07 | 7 .23 | 10 .93 |
|  | 6D | AX-95003520 | 467 | 8 .00E-03 | 4 .82 | 5 .53 |
|  | 7B | AX-94505411 | 618 | 4 .73E-02 | -3 .02 | 4 .64 |
|  | UN | RAC875_c67770_822 |  | 2 .43E-03 | -3 .41 | 6 .28 |
| Keny 2021 | 2A | wsnp_Ex_c16627_25162391 | 717 | 1 .68E-02 | -3 .58 | 6 .04 |
|  | 2D | PpdDD001 | 34 | 3 .74E-02 | 2 .72 | 5 .39 |
| Tolu 2021 | 6B | AX-94549612 | 710 | 4 .69E-07 | 6 .63 | 10 .60 |
|  | 7D | TA007516-0910 | 597 | 1 .52E-02 | 4 .61 | 5 .79 |
| PX 2021 | 1B | wsnp_Ex_rep_c67299_65844168 | 586 | 2.46E-02 | -6.91 | 4.97 |
|  | 2B | wsnp_Ex_c20169_29215401 | 554 | 3.18E-06 | 6.53 | 9.77 |
|  | 2B | BS00067907_51 | 722 | 5.35E-03 | 5.25 | 5.94 |
|  | 3A | AX-158577637 | 7 | 2.28E-02 | 4.34 | 5.07 |
|  | 3D | AX-94922201 | 553 | 8.43E-03 | 6.28 | 5.56 |
|  | 5B | AX-108953209 | 515 | 3.12E-04 | -8.07 | 7.47 |
|  | 5B | BS00049213_51 | 590 | 3.69E-03 | 5.31 | 6.23 |
|  | 5D | Kukri_c14692_75 | 548 | 5.55E-03 | 5.33 | 5.83 |
| XD 2021 | 1B | Tdurum_contig65853_242 | 629 | 4.55E-02 | 5.08 | 4.83 |
|  | 2A | AX-94482613 | 729 | 3.46E-02 | -5.69 | 5.43 |
|  | 2B | AX-94833805 | 711 | 1.41E-03 | -6.37 | 7.12 |
|  | 4A | Kukri_rep_c109463_264 | 631 | 3.64E-02 | -3.62 | 5.01 |
|  | 5D | Kukri_c14692_75 | 548 | 3.46E-02 | 5.00 | 5.13 |
|  | 7A | RAC875_c9700_989 | 671 | 3.46E-02 | 6.69 | 5.19 |
